# Research and Remedies

**Published:** 1913-05-17

**Authors:** 


### RESEARCH AND REMEDIES.

### The "Jigger" in South Africa.

Readers of Waterton or Bates, or any other traveller in tropical South America, will not need to be told that those regions were the original home of a pest long known as the jigger flea, though the more correct form is the chigoe. Within the last thirty years or so this creature has appeared in Africa, first on the west coast, and eventually in most of the tropical parts of the continent, which are at all thickly populated. Now, we learn from an article in the *Transvaal Medical Journal*, it has turned up in the Middelburg district of the Transvaal, to spread, doubtless, far and wide through the Union. This animal is much more than a source of mild annoyance, such as its relatives which inhabit Europe are; it is a source of endless expense and suffering, even indirectly of death sometimes. Its peculiarity is that it attaches itself by preference to the feet, and hence attacks mainly those who go habitually barefoot—that is, the natives. The gravid female burrows into the skin, and causes no more than a slight itching in the process. Thus, she may secure a nest for herself without her victim having any knowledge of the fact. Here she produces her young, and the result is a painful abscess, only to be cured by the complete evacuation of the jigger and her family. Sometimes she even manages to get under the toe-nail, in which case the consequences are even more painful and disabling than when she chooses the under surface of the toe. White people also may harbour these unpleasant parasites; and if, as seems likely, the jigger spreads through South Africa, it is absolutely certain that Europeans will not escape. Children are especially likely to suffer, owing to their fondness for running about barefooted.

### A Satisfactory Case.

The resources of modern surgery as applied to the relief of desperate diseases are well illustrated by a case recorded in the *Edinburgh Medical Journal* by Mr. Alexis Thomson. In 1906 a girl of nineteen developed a tumour in the upper part of the thigh which was removed by a surgeon. For three and a half years she remained well, but then a recurrence took place. As the growth caused no pain she did not resort again to surgery for eighteen months, during which time the mass got steadily larger, until it caused discomfort in walking. It was dusky red in colour, and the inguinal glands were enlarged and hard. Mr. Thomson thought it just possible to remove the whole disease by amputating through the upper end of the femur, and proceeded to do so. First he opened the abdomen to see the condition of the lumbar glands; these were free from obvious growth, so the common iliac artery was tied, the abdomen closed, and the operation proceeded with. The growth turned out to be a fibro-sarcoma, but the enlarged glands were not infected with it. The following year the patient returned on account of a mild ventral hernia; this was operated on and cured, and the opportunity was taken of inspecting the iliac artery, which was found entirely obliterated. In April 1913, two years after the amputation, the patient is in perfect health and activity.

